# Surgical Interventions in Chronic Pancreatitis: A Systematic Review of Their Impact on Quality of Life

**DOI:** 10.7759/cureus.53989

**Published:** 2024-02-10

**Authors:** Abdullah Ashfaq, Nikhil Deep Kolanu, Mathani Mohammed, Sergio Rodrigo Oliveira Souza Lima, Abdur Rehman, Abdullah Shehryar, Nader A Fathallah, Shenouda Abdallah, Ismail S Abougendy, Ali Raza

**Affiliations:** 1 Surgery, Gujranwala Medical Teaching Hospital, Gujranwala, PAK; 2 Internal Medicine, China Medical University, Shenyang, CHN; 3 General Surgery, Sudan Medical Specialization Board Hospital, Khartoum, SDN; 4 Plastic Surgery, Hospital da Bahia, Salvador, BRA; 5 Surgery, Mayo Hospital, Lahore, PAK; 6 Internal Medicine, Allama Iqbal Medical College, Lahore, PAK; 7 Accident and Emergency, Nasr City Hospital for Health Insurance, Cairo, EGY; 8 Surgery, Sheikh Jaber Al-Ahmad Al-Sabah Hospital, Kuwait City, KWT; 9 General Surgery, Misr University for Science and Technology, Cairo, EGY; 10 Internal Medicine, Nishtar Medical University, Multan, PAK

**Keywords:** pancreatitis, pancreatic procedures, systematic review, quality of life, surgery, chronic pancreatitis

## Abstract

This systematic review evaluates the efficacy of surgical interventions in improving the quality of life for patients with chronic pancreatitis (CP). A thorough literature search, following Preferred Reporting Items for Systematic Reviews and Meta-Analyses (PRISMA) guidelines, identified 11 studies that focused on patient-reported outcomes after surgical treatments, including pancreatic resections, drainage procedures, and duodenum-preserving head resections. The findings indicate that organ-preserving procedures, notably the Frey and Beger operations, significantly enhance pain control and overall quality of life while reducing analgesic dependency. This review provides crucial insights into the long-term efficacy and comparative benefits of different surgical approaches, highlighting the need for personalized surgical strategies in CP management. It emphasizes the necessity for standardized outcome measures and further comparative research to refine CP treatment protocols.

## Introduction and background

Pancreatitis, in its acute and chronic forms, is a significant health concern globally. Acute pancreatitis is typically a sudden and severe onset of the disease, often linked with gallstones or alcohol use, and can range from mild discomfort to life-threatening complications. Chronic pancreatitis (CP), in contrast, is a long-standing inflammation of the pancreas that leads to permanent structural damage. CP represents a progressive inflammatory disorder characterized by irreversible destruction of the pancreatic parenchyma, culminating in both exocrine and endocrine insufficiencies [1]. This condition significantly impairs health-related quality of life (HRQOL), predominantly manifesting through symptoms such as recurrent or persistent abdominal pain, steatorrhea, weight loss, and diabetes mellitus [2]. In Western countries, excessive alcohol consumption is identified as the primary etiological factor in 60-90% of CP cases. Other notable causative factors include genetic mutations and autoimmune processes [3].

The global incidence of CP is estimated to be between 4 and 12 per 100,000 individuals. However, in regions with high incidence rates, such as India, the incidence soars to 114-200 per 100,000 population, primarily attributed to non-alcoholic factors. Non-alcoholic causes of chronic pancreatitis include genetic mutations, autoimmune disorders, and structural abnormalities of the pancreatic ducts. Other risk factors, such as chronic hypercalcemia, hyperlipidemia, and certain medications can also contribute to the development of CP. Recurrent episodes of acute pancreatitis, regardless of the cause, can evolve into chronic pancreatitis over time. Environmental factors like exposure to certain toxins or high levels of oxidative stress are also implicated in non-alcoholic CP. The prevalence of CP ranges from 26 to 46 cases per 100,000 population [4]. The disease burden of CP extends beyond physical symptoms, significantly affecting patients' social-emotional well-being and often leading to recurrent hospitalizations, work disability, and opioid dependency [5].

Management strategies for CP primarily focus on alleviating pain, replacing exocrine and endocrine functions, addressing local complications, promoting cessation of alcohol and smoking, and implementing dietary modifications [2]. Endoscopic therapies, as a part of medical management, play a significant role in the treatment of CP, particularly in managing pain and complications. These therapies include endoscopic pancreatic duct drainage, stent placement, and lithotripsy, which are aimed at relieving ductal obstructions and reducing pancreatic ductal hypertension, a common source of pain in CP patients. Endoscopic ultrasound-guided therapies have also been developed for targeted celiac plexus blocks or neurolysis, providing an option for pain relief. Despite the utilization of these optimal medical and endoscopic therapies, a substantial proportion of patients, ranging from 40-75%, eventually necessitate surgical intervention. This requirement is often due to intractable pain or complications such as biliary obstruction and duodenal stenosis [6]. The use of endoscopic treatments can be a valuable intermediary step before considering surgery, and in some cases, can delay or even prevent the need for surgical intervention. However, their effectiveness varies and some patients do not achieve lasting relief, necessitating more invasive approaches. The primary objectives of surgical intervention include achieving sustained pain relief, managing complications, striving to preserve pancreatic function, and enhancing long-term HRQOL [7].

The decision-making process regarding the most suitable surgical approach is intricate, owing to the disease's varied morphological manifestations and the plethora of available resection and drainage procedures [8]. Traditional techniques, such as pancreaticoduodenectomy or distal pancreatectomy, which involve sectioning of the pancreas, have been widely practiced. However, these procedures are often linked with suboptimal pain relief outcomes and a progressive decline in pancreatic function over time [9]. In contrast, duodenum-preserving pancreatic head resections, exemplified by the Frey or Beger procedure, especially when combined with drainage techniques, have demonstrated superior outcomes [8]. Nonetheless, a need exists for comparative studies that rigorously evaluate the impact of different surgical methods on the quality of life, underscoring a significant gap in current medical research.

The primary objective of our systematic review was to meticulously evaluate the effectiveness of various surgical interventions for chronic pancreatitis (CP) in improving patient-reported quality of life (QoL). This entails a comprehensive analysis of randomized controlled trials and observational studies to assess the impact of these surgical techniques on short-term and long-term health-related QoL. We aimed to identify and compare the outcomes of different surgical strategies, focusing on their efficacy in pain management, preservation of pancreatic function, and overall enhancement of patients' physical, social, and emotional well-being. By doing so, we seek to provide clinicians with a robust, evidence-based guide in selecting the most appropriate surgical approach for CP patients, thereby contributing to improved patient care and outcomes in this complex medical condition.

## Review

Materials and methods

Search Strategy

In our systematic review investigating the impact of surgical procedures on the quality of life in patients with chronic pancreatitis, we meticulously developed a search strategy that aligns with Preferred Reporting Items for Systematic Reviews and Meta-Analyses (PRISMA) guidelines. Our approach involved a comprehensive search across several esteemed databases as follows: PubMed, Embase, Cochrane Library, and Web of Science. This database selection aimed to capture a wide array of relevant scientific literature. Key to our strategy was the careful selection of specific keywords and phrases, such as "chronic pancreatitis," "surgical procedures," "quality of life," "life improvement," "pain management," and "pancreatic surgery." These terms were chosen to encapsulate our research question thoroughly. To optimize the search, we employed Boolean operators like "AND" and "OR," which allowed us to create effective combinations of these keywords. We set our search time frame from the inception of each database up to December 2023, aiming to include all relevant and up-to-date research. This structured and detailed search strategy enabled us to meticulously explore and analyze numerous studies, laying a solid foundation for our systematic review. This review is intended to shed light on the various surgical interventions and their effects on the quality of life for patients suffering from chronic pancreatitis, thereby offering valuable insights to the medical community.

Eligibility Criteria

For our systematic review focusing on the efficacy of surgical procedures in improving the quality of life for patients with chronic pancreatitis, we have carefully delineated our eligibility criteria to ensure thoroughness and relevance. Our inclusion criteria are multifaceted and specifically tailored. We are primarily considering peer-reviewed research articles, cohort studies, and clinical trials, as these sources are rich in high-quality, evidence-based research essential for an exhaustive review. The target population for this review is patients diagnosed with chronic pancreatitis, ensuring that our research remains focused on the pertinent group. Studies that specifically examine the impact of various surgical interventions on the quality of life in these patients are included. This encompasses research that evaluates the outcomes of different surgical techniques, their efficacy in pain management, and their overall impact on patients' quality of life and daily functioning. To ensure linguistic uniformity and comprehensibility, we include studies published in English, the predominant language in scientific discourse.

On the other hand, our exclusion criteria are defined to maintain the focus and precision of the review. To keep our review targeted and relevant, we will exclude studies that do not directly relate to surgical interventions in chronic pancreatitis or do not assess quality-of-life outcomes. Research based solely on animal models is excluded, as our primary aim was to extract data pertinent to human subjects. Non-English language publications will not be considered due to potential challenges in translation and interpretation. Additionally, to ensure the reliability and validity of our review, we excluded gray literature, such as conference abstracts, posters, and unpublished manuscripts. Studies that need comprehensive data or do not address the research question adequately were also excluded, as they might compromise our findings' overall quality and reliability.

This carefully structured eligibility criterion was designed to guide the selection of studies, ensuring that our systematic review is comprehensive and focused on providing meaningful insights into the surgical management of chronic pancreatitis and its impact on a patient's quality of life.

Data Extraction

For our systematic review assessing surgical procedures for enhancing the quality of life in patients with chronic pancreatitis, we employed a detailed and systematic data extraction approach to guarantee the reliability and validity of our results. This process was executed in two distinct stages to ensure thoroughness and accuracy.

In the initial stage, our focus was on preliminary screening based on the titles and abstracts of articles. This initial screening was instrumental in identifying articles potentially relevant to our research topic. We engaged two independent reviewers to examine the abstracts meticulously. Their task was to evaluate the relevance of each article, categorizing them as "relevant," "not relevant," or "possibly relevant." This categorization was based on a careful and systematic assessment, ensuring that each article was appropriately classified according to its pertinence to our research question.

The second stage of our data extraction process entailed a more detailed review. Here, the full-text articles deemed potentially eligible were thoroughly examined. Two independent reviewers were tasked with extracting data from these articles, utilizing a standardized data extraction form created in Microsoft Excel. This form was designed to capture a wide range of critical information systematically. Each reviewer independently applied our predefined inclusion and exclusion criteria to these studies. In cases where discrepancies arose between the two primary reviewers, we involved a third reviewer. This reviewer independently assessed the articles to resolve disagreements through discussion and consensus. This step was crucial in ensuring the data extraction process's accuracy and consistency and the studies' eligibility assessment.

The data extraction form was comprehensive and designed to collect vital information from each study. This included the author's names, characteristics of study participants, study setting, study design, types of surgical interventions, outcome measures, and key findings. This meticulous data collection process enabled a detailed and nuanced analysis of the studies' findings. It ensured that all relevant information was captured and considered in our systematic review, enhancing our analysis's depth and breadth.

 Data Analysis and Synthesis

In our systematic review, we employed a comprehensive approach for the analysis and synthesis of data focusing on the impact of surgical procedures on the quality of life in patients with chronic pancreatitis. Our data analysis and synthesis methodology was primarily narrative and aimed at providing a detailed and contextual understanding of the research findings.

Given the complexity and heterogeneity of the surgical procedures and patient outcomes, a meta-analysis was deemed unfeasible. Instead, we opted for a narrative synthesis, which allowed us to explore and interpret the data in a manner that captures the nuances and variations across different studies. This approach was particularly suitable for accommodating the diverse methodologies, interventions, and outcome measures reported in the studies.

Our narrative synthesis involved systematically collating and summarizing the key findings from each study. We focused on identifying patterns, themes, and relationships within the data, particularly those relating to the types of surgical interventions, their effectiveness in improving the quality of life, and any associated complications or adverse outcomes. We also paid special attention to variations in study designs, populations, and geographical contexts to understand how these factors might influence the outcomes.

The analysis was conducted with an emphasis on critically evaluating the strengths and limitations of each study, thereby providing a balanced and comprehensive view of the current evidence. We assessed the methodological quality of the studies and considered how this impacted the reliability and applicability of their findings.

Through this narrative synthesis, we aimed to draw meaningful conclusions about the efficacy of different surgical procedures in enhancing the quality of life for patients with chronic pancreatitis. The synthesis provided insights into the most effective surgical practices and highlighted areas where evidence was lacking, thus identifying gaps in the current research and suggesting directions for future studies. This narrative approach enabled us to create a nuanced and detailed understanding of a complex and multifaceted area of medical research.

Results 

Study Selection Process

Our study selection process for the systematic review of surgical procedures for improving the quality of life in patients with chronic pancreatitis was scrupulously conducted following the PRISMA guidelines. The process began with an expansive search across several databases, initially identifying 760 records. To refine our search, we removed 23 duplicate records, resulting in 737 studies that were screened for relevance. Titles and abstracts were reviewed, excluding 354 records not meet the preliminary relevance criteria.

From the remaining 383 reports, we sought to retrieve the full texts for a more detailed evaluation. However, we needed help to retrieve 341 reports, which left us with 42 full-text articles to assess for eligibility. This rigorous assessment examined the study design, population, interventions, and outcomes relevant to our review. After this thorough process, 31 reports were excluded for various reasons, such as lack of relevance to the surgical improvement of quality of life in chronic pancreatitis or failure to meet the inclusion criteria's methodological standards.

**Figure 1:**
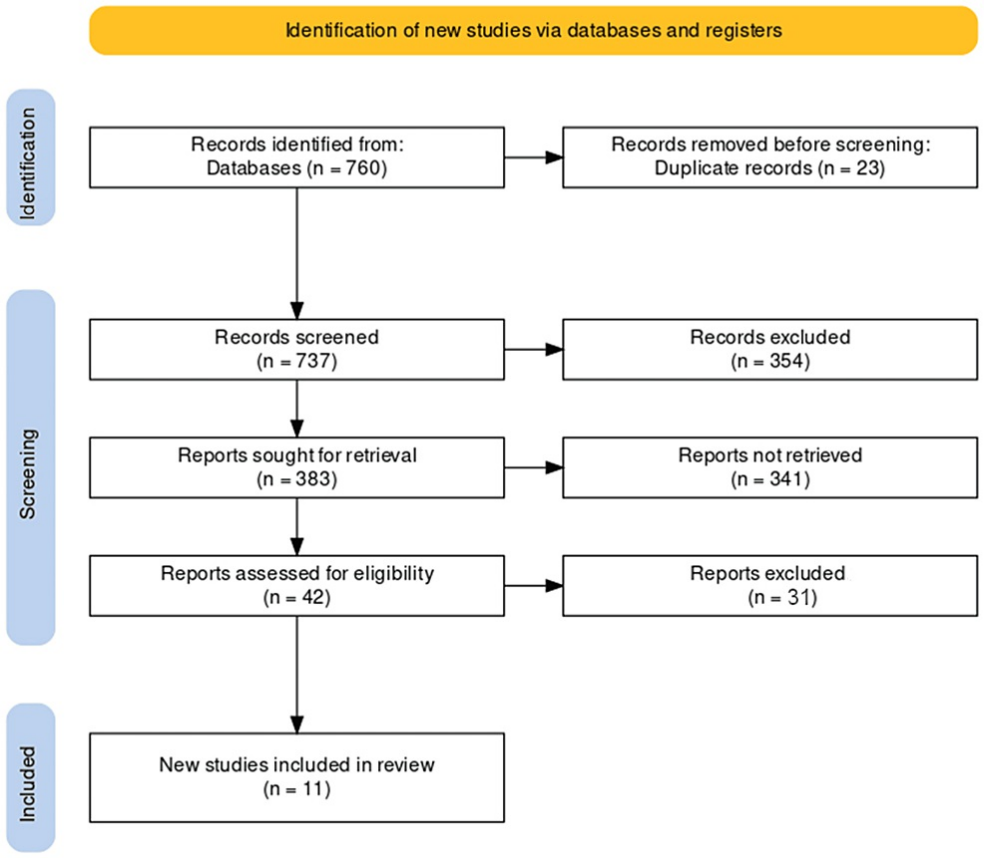
PRISMA flow diagram of the selection of studies for inclusion in the systematic review. PRISMA: Preferred Reporting Items for Systematic Reviews and Meta-Analyses

This diligent selection process led to the inclusion of 11 studies in our review. These studies were identified as the most relevant and provided a comprehensive evidence base for analysis in our systematic review. Each step of our selection process was documented in detail, and the flow of information through the different phases of the study selection process can be visualized in the accompanying PRISMA flowchart (Figure 1).

Characteristics of Selected Studies

In our systematic review focusing on the impact of surgical procedures on the quality of life in patients with chronic pancreatitis, 11 studies met the stringent inclusion criteria we established. The selected studies comprised diverse research designs, including longitudinal studies, prospective and retrospective analyses, randomized clinical trials, and prospective observational studies. This variety in study designs provided a comprehensive overview of the topic from different research perspectives.

These studies were conducted geographically across various countries, reflecting a global perspective on managing chronic pancreatitis. The majority were conducted in European and North American countries, with contributions from Germany, the United States, and Italy. This geographical diversity is crucial as it adds to the generalizability of our findings across different healthcare settings.

The characteristics and main findings of the included studies are summarized in Table 1. This table serves as a concise reference point for the key aspects of each study, including the authors, study design, sample size, type of intervention, primary outcomes measured, and the principal findings. This summary aimed to provide a clear and structured overview of the research landscape regarding surgical interventions for chronic pancreatitis and their impact on patient's quality of life.

**Table 1:** Summary of key studies assessing the efficacy of surgical interventions in chronic pancreatitis. DPPHR: duodenum-preserving pancreatic head resection; CP: chronic pancreatitis; QoL: quality of life; LPJ-LPHE: longitudinal pancreaticojejunostomy combined with local pancreatic head excision; PPPD: pylorus-preserving pancreatoduodenectomy; EORTC QLQ-C30: European Organization for Research and Treatment of Cancer Quality of Life Questionnaire C30; VAS: visual analog scale; TPIAT: total pancreatectomy with islet autotransplantation

Studies	Objective	Study design	Sample size	Intervention	Outcomes	Key findings
Beger et al. [10]	To present preoperative and early postoperative data for DPPHR in severe CP	Longitudinal, up to 26 years	504 patients	DPPHR	Pain status, hospital admission, endocrine function, rehabilitation, and QoL	78.8% pain-free after up to 14 years
Izbicki et al. [11]	Compare duodenum-preserving resection techniques in CP	Prospective, randomized	42 patients	Beger’s vs. Frey’s procedures	Morbidity, pain relief, QoL, and organ function	No mortality, 14% morbidity
Hildebrand et al. [12]	Influence of surgery on CP and postoperative QoL	Retrospective, prospectively collected data	51 patients	Frey and Whipple procedures	Pain scores, QoL, and morbidity rates	92.3% of the Frey group improved pain scores
Izbicki et al. [13]	Efficacy of LPJ-LPHE vs. PPPD in CP	Prospective, randomized	61 patients	LPJ-LPHE vs. PPPD	In-hospital complications, pain score, and QoL	94% pain score decrease in LPJ-LPHE
Gopalakrishnan et al. [14]	Compare Frey’s plus and only Frey’s procedure in CP	Retrospective database analysis	100 patients	Frey’s plus vs. only Frey’s	Pain relief, QoL, and operative parameters	Common additional procedure: Roux-en-Y hepaticojejunostomy
Strate et al. [15]	Long-term comparison of Beger and Frey procedures in CP	Long-term follow-up, randomized trial	74 patients	Beger vs. Frey	Mortality, QoL, pain, and organ function.	No difference in mortality, QoL, or pain
Aimoto et al. [16]	Short- and long-term outcomes of Frey's vs. PPPD in CP.	Retrospective patient outcomes	16 patients	Frey's vs. PPPD	Pain, QoL, physical status, complications, and organ function	Significant pain reduction and QoL improvement post surgery
Jeppe et al. [17]	Evaluate post Frey procedure QoL in CP	Prospective, observational, longitudinal	32 patients	Frey procedure	QoL using EORTC QLQ-C30 and structured interview	Clinically relevant improvements in QoL postoperatively
Rath et al. [18]	The outcome of Frey’s procedure in CP in terms of QoL	Prospective observational	35 patients	Frey’s procedure	QoL using VAS score and EORTC QLQ-C30	Significant improvements in all QoL domains postoperatively
Kerremans et al. [19]	Effectiveness of subtotal resection and ductal obliteration in CP	Retrospective patient outcomes	12 patients	Subtotal resection and ductal obliteration	Pain relief, pseudocyst formation, hyperglycemia, and QoL.	Lasting pain relief in 10 patients
Bellin et al. [20]	Recommendations on TPIAT for CP complications	Review and consensus meeting	N/A	TPIAT evaluation	Indications for TPIAT, optimal timing, team roles, and long-term management	TPIAT is effective in managing CP complications and cancer risk

Discussion

Our systematic review provides a detailed understanding of surgical interventions in chronic pancreatitis (CP), informed by data from 11 individual studies. These studies illuminate the impacts of these surgeries in the short and long term across physical, social, and emotional aspects of health-related quality of life (QoL).

A pivotal study by Beger et al. showcases the long-term efficacy of duodenum-preserving pancreatic head resection (DPPHR), with 78.8% of patients reporting pain-free up to 14 years post surgery [10]. This outcome is crucial in demonstrating the lasting benefits of DPPHR in severe cases of CP. The success of DPPHR also suggests that preserving pancreatic tissue can be a key factor in managing CP effectively while maintaining patient quality of life.

The research by Izbicki et al., which compares various resection techniques, is significant [11,13]. Their findings show minimal mortality and a 14% morbidity rate when comparing Beger's and Frey's procedures [11]. Another study from the same authors emphasizes the effectiveness of the longitudinal pancreaticojejunostomy combined with local pancreatic head excision (LPJ-LPHE) vs. the pylorus-preserving pancreatoduodenectomy (PPPD), indicating a considerable reduction in pain scores post surgery [13]. This highlights the evolving nature of surgical techniques and the importance of tailoring interventions to the individual needs of patients.

Gopalakrishnan et al. provide a comparative analysis between Frey's plus and only Frey's procedures, offering insights into how adjunct procedures can augment outcomes, especially regarding pain relief and QoL [14]. This stresses the need for customized surgical approaches in managing CP, further reinforcing the concept of personalized medicine in surgical decision-making.

Strate et al. contributed a long-term follow-up study comparing the Beger and Frey procedures, showing similar outcomes in mortality, QoL, and pain relief [15]. This suggests choosing between these techniques should be based on individual patients' clinical profiles. The retrospective analysis by Aimoto et al. comparing Frey's and PPPD procedures highlights significant improvements in pain and QoL post surgery, supporting the effectiveness of these interventions [16].

The Frey and Beger procedures have marked a significant advancement in the quality of life for patients with chronic pancreatitis. The Frey procedure, combining local resection with lateral pancreaticojejunostomy, has been particularly effective in alleviating pain and improving daily functioning, a key factor in patient well-being. Similarly, the Beger procedure, with its duodenum-preserving resection, not only mitigates pain but also preserves organ function, leading to better postoperative outcomes. Both techniques signify a shift towards patient-centered care in chronic pancreatitis management, showing consistent improvements in patients' ability to engage in daily activities and overall life satisfaction [15,16].

Jeppe et al. and Rath et al., focusing on Frey's procedure, utilize the European Organization for Research and Treatment of Cancer Quality of Life Questionnaire C30 (EORTC QLQ-C30) tool, revealing significant postoperative improvements in QoL [17,18]. This underscores the procedure's role in enhancing patient well-being.

Kerremans et al. and Bellin et al. explore less conventional techniques like subtotal resection and ductal obliteration, and total pancreatectomy with islet autotransplantation (TPIAT), respectively, highlighting the range of surgical options for CP management [19,20]. These studies collectively affirm the clinical benefits of surgical intervention in CP, especially in refractory cases. They spotlight the success of techniques, such as pancreatic resections [21], drainage procedures [22], and duodenum-preserving head resections in improving pain management, reducing reliance on analgesics, and enhancing overall QoL [23].

Particularly effective are organ-preserving strategies, namely the Frey [24] and Beger [25] operations, corroborated by evidence from five randomized controlled trials (RCTs) [8,23,26-28] and pooled data from over 2000 patients in 16 case series [9,29-31]. Nonetheless, the heterogeneity in reporting QoL outcomes and the variability in follow-up durations are notable limitations in the current literature [29,32]. These factors call for cautious interpretation of results and underscore the need for standardized reporting in future research.

Our review, enriched by individual study analyses, delivers in-depth insights into the surgical management of CP. It emphasizes the significance of personalized surgical approaches and the potential for considerable improvements in QoL for CP patients. These findings are crucial for guiding clinical decision-making and future research in this complex and challenging medical field. As the field of pancreatic surgery continues to evolve, ongoing research and innovation are essential to refine surgical techniques and enhance patient outcomes further.

## Conclusions

This systematic review provides a critical analysis of the efficacy of surgical interventions in chronic pancreatitis (CP), emphasizing their impact on patient-reported quality of life (QoL). Our findings highlight that organ-preserving procedures, notably the Frey and Beger operations, significantly improve pain management and overall QoL in CP patients. This review is a valuable guide for clinicians in selecting appropriate surgical strategies tailored to individual patient needs. It also identifies gaps in current research, such as the need for standardized QoL outcome measures and comparative studies, underscoring the importance of ongoing investigation in this field. Ultimately, this work reinforces the significance of personalized surgical approaches in enhancing the quality of life for patients with CP.

## References

[1] Ito T, Ishiguro H, Ohara H (2016). Evidence-based clinical practice guidelines for chronic pancreatitis 2015. J Gastroenterol.

[2] Löhr JM, Dominguez-Munoz E, Rosendahl J (2017). United European Gastroenterology evidence-based guidelines for the diagnosis and therapy of chronic pancreatitis (HaPanEU). United European Gastroenterol J.

[3] Yadav D, Lowenfels AB (2013). The epidemiology of pancreatitis and pancreatic cancer. Gastroenterology.

[4] Machicado JD, Yadav D (2017). Epidemiology of recurrent acute and chronic pancreatitis: similarities and differences. Dig Dis Sci.

[5] Rückert F, Distler M, Hoffmann S (2011). Quality of life in patients after pancreaticoduodenectomy for chronic pancreatitis. J Gastrointest Surg.

[6] Ahmed Ali U, Issa Y, Bruno MJ (2013). Early surgery versus optimal current step-up practice for chronic pancreatitis (ESCAPE): design and rationale of a randomized trial. BMC Gastroenterol.

[7] Ke N, Jia D, Huang W, Nunes QM, Windsor JA, Liu X, Sutton R (2018). Earlier surgery improves outcomes from painful chronic pancreatitis. Medicine (Baltimore).

[8] Strate T, Bachmann K, Busch P (2008). Resection vs drainage in treatment of chronic pancreatitis: long-term results of a randomized trial. Gastroenterology.

[9] Jawad ZA, Tsim N, Pai M, Bansi D, Westaby D, Vlavianos P, Jiao LR (2016). Short and long-term post-operative outcomes of duodenum preserving pancreatic head resection for chronic pancreatitis affecting the head of pancreas: a systematic review and meta-analysis. HPB (Oxford).

[10] Beger HG, Schlosser W, Friess HM, Büchler MW (1999). Duodenum-preserving head resection in chronic pancreatitis changes the natural course of the disease: a single-center 26-year experience. Ann Surg.

[11] Izbicki JR, Bloechle C, Knoefel WT, Kuechler T, Binmoeller KF, Broelsch CE (1995). Duodenum-preserving resection of the head of the pancreas in chronic pancreatitis. A prospective, randomized trial. Ann Surg.

[12] Hildebrand P, Duderstadt S, Jungbluth T, Roblick UJ, Bruch HP, Czymek R (2011). Evaluation of the quality of life after surgical treatment of chronic pancreatitis. J Pancreas.

[13] Izbicki JR, Bloechle C, Broering DC, Knoefel WT, Kuechler T, Broelsch CE (1998). Extended drainage versus resection in surgery for chronic pancreatitis: a prospective randomized trial comparing the longitudinal pancreaticojejunostomy combined with local pancreatic head excision with the pylorus-preserving pancreatoduodenectomy. Ann Surg.

[14] Gopalakrishnan G, Kalayarasan R, Gnanasekaran S, Pottakkat B (2020). Frey's plus versus Frey's procedure for chronic pancreatitis: analysis of postoperative outcomes and quality of life. Ann Hepatobiliary Pancreat Surg.

[15] Strate T, Taherpour Z, Bloechle C (2005). Long-term follow-up of a randomized trial comparing the beger and frey procedures for patients suffering from chronic pancreatitis. Ann Surg.

[16] Aimoto T, Uchida E, Matsushita A, Kawano Y, Mizutani S, Kobayashi T (2013). Long-term outcomes after Frey's procedure for chronic pancreatitis with an inflammatory mass of the pancreatic head, with special reference to locoregional complications. J Nippon Med Sch.

[17] Jeppe CY, Becker P, Smith MD (2013). Post-Frey procedure quality of life in South African patients with painful chronic pancreatitis. JOP.

[18] Rath S, Meher S, Basu A, Priyadarshini S, Rout B, Sharma R (2016). Quality of life after Frey’s procedure in patients with chronic pancreatitis. J Clin Diagn Res.

[19] Kerremans RP, Penninckx FM, De Groote J, Fevery J (1987). Subtotal resection of the head of the pancreas combined with ductal obliteration of the distal pancreas in chronic pancreatitis. Ann Surg.

[20] Bellin MD, Freeman ML, Gelrud A (2014). Total pancreatectomy and islet autotransplantation in chronic pancreatitis: recommendations from PancreasFest. Pancreatology.

[21] Jimenez RE, Fernandez-del Castillo C, Rattner DW, Chang Y, Warshaw AL (2000). Outcome of pancreaticoduodenectomy with pylorus preservation or with antrectomy in the treatment of chronic pancreatitis. Ann Surg.

[22] Cahen DL, Gouma DJ, Nio Y (2007). Endoscopic versus surgical drainage of the pancreatic duct in chronic pancreatitis. N Engl J Med.

[23] Klempa I, Spatny M, Menzel J, Baca I, Nustede R, Stöckmann F, Arnold W (1995). [Pancreatic function and quality of life after resection of the head of the pancreas in chronic pancreatitis. A prospective, randomized comparative study after duodenum preserving resection of the head of the pancreas versus Whipple's operation]. Chirurg.

[24] Farkas G, Leindler L, Daróczi M, Farkas G Jr (2003). Organ-preserving pancreatic head resection in chronic pancreatitis. Br J Surg.

[25] Büchler MW, Friess H, Müller MW, Wheatley AM, Beger HG (1995). Randomized trial of duodenum-preserving pancreatic head resection versus pylorus-preserving Whipple in chronic pancreatitis. Am J Surg.

[26] Witzigmann H, Max D, Uhlmann D (2003). Outcome after duodenum-preserving pancreatic head resection is improved compared with classic Whipple procedure in the treatment of chronic pancreatitis. Surgery.

[27] Farkas G, Leindler L, Daróczi M, Farkas G Jr (2006). Prospective randomised comparison of organ-preserving pancreatic head resection with pylorus-preserving pancreaticoduodenectomy. Langenbecks Arch Surg.

[28] Diener MK, Rahbari NN, Fischer L, Antes G, Büchler MW, Seiler CM (2008). Duodenum-preserving pancreatic head resection versus pancreatoduodenectomy for surgical treatment of chronic pancreatitis: a systematic review and meta-analysis. Ann Surg.

[29] Keck T, Wellner UF, Bahra M (2016). Pancreatogastrostomy versus pancreatojejunostomy for reconstruction after pancreatoduodenectomy (RECOPANC, DRKS 00000767): perioperative and long-term results of a multicenter randomized controlled trial. Ann Surg.

[30] Huang JJ, Yeo CJ, Sohn TA (2000). Quality of life and outcomes after pancreaticoduodenectomy. Ann Surg.

[31] Navadgi S, Pandanaboyana S, Windsor JA (2015). Surgery for acute pancreatitis. Indian J Surg.

[32] Gooiker GA, van Gijn W, Wouters MW, Post PN, van de Velde CJ, Tollenaar RA (2011). Systematic review and meta-analysis of the volume-outcome relationship in pancreatic surgery. Br J Surg.

